# Upregulation of Krebs cycle and anaerobic glycolysis activity early after onset of liver ischemia

**DOI:** 10.1371/journal.pone.0199177

**Published:** 2018-06-14

**Authors:** Tom S. Chan, Shamir Cassim, Valérie-Ann Raymond, Sven Gottschalk, Grégory Merlen, Claudia Zwingmann, Pascal Lapierre, Peter Darby, Cyril David Mazer, Marc Bilodeau

**Affiliations:** 1 Laboratoire d’hépatologie cellulaire, Centre de recherche du Centre hospitalier de l’Université de Montréal, Montréal, Québec, Canada; 2 Neuroscience Research Unit, Hôpital Saint-Luc, Université de Montréal, Québec, Canada; 3 Département de médecine, Université de Montréal, Montréal, Québec, Canada; 4 Department of Anesthesia, Keenan Research Centre in the Li Ka Shing Knowledge Institute of Saint Michael’s Hospital, University of Toronto, Toronto, Ontario, Canada; University of Navarra School of Medicine and Center for Applied Medical Research (CIMA), SPAIN

## Abstract

The liver is a highly vascularized organ receiving a dual input of oxygenated blood from the hepatic artery and portal vein. The impact of decreased blood flow on glucose metabolism and how hepatocytes could adapt to this restrictive environment are still unclear. Using the left portal vein ligation (LPVL) rat model, we found that cellular injury was delayed after the onset of liver ischemia. We hypothesized that a metabolic adaptation by hepatocytes to maintain energy homeostasis could account for this lag phase. Liver glucose metabolism was characterized by ^13^C- and ^1^H-NMR spectroscopy and analysis of high-energy metabolites. ALT levels and caspase 3 activity in LPVL animals remained normal during the first 12 h following surgery (*P*<0.05). Ischemia rapidly led to decreased intrahepatic tissue oxygen tension and blood flow (*P*<0.05) and increased expression of Hypoxia-inducible factor 1-alpha. Intrahepatic glucose uptake, ATP/ADP ratio and energy charge level remained stable for up to 12 h after ligation. Entry of glucose in the Krebs cycle was impaired with lowered incorporation of ^13^C from [U^-13^C]glucose into glutamate and succinate from 0.25 to 12 h after LPVL. However, total hepatic succinate and glutamate increased 6 and 12 h after ischemia (*P*<0.05). Glycolysis was initially reduced (*P*<0.05) but reached maximum ^13^C-lactate (*P*<0.001) and ^13^C-alanine (*P*<0.01) enrichments 12 h after LPVL. In conclusion, early liver homeostasis stems from an inherent ability of ischemic hepatocytes to metabolically adapt through increased Krebs cycle and glycolysis activity to preserve bioenergetics and cell viability. This metabolic plasticity of hepatocytes could be harnessed to develop novel metabolic strategies to prevent ischemic liver damage.

## Introduction

The liver plays a central role in metabolic homeostasis and is a major site for synthesis, metabolism, storage and redistribution of carbohydrates, proteins and lipids [1, 2]. The liver is a highly vascularized organ that receives 25% of the cardiac input through a dual input via the hepatic artery and the portal vein that are responsible for 25% and 75% of the liver flow respectively. The liver displays a remarkable capacity to resist to ischemic insults and to nutrient deprivation. Nevertheless, a variety of pathological conditions have been characterized by an ischemic insult to the liver: it is therefore of significant interest to understand how hepatocytes respond, adapt and resist to these conditions [3–6].

Liver ischemic injury is of paramount importance in organ transplantation [7] but is also of clinical significance in cirrhosis [8], thrombosis [9] and Budd-Chiari syndrome [10]. Ischemic insult and inflammatory-mediated injury can occur during organ harvesting, in the peritransplant period and after revascularization [11]. Liver hypoxia and ischemia have a strong impact on liver cell homeostasis and function leading to hepatocyte metabolic alterations that can ultimately result in cell death through both apoptosis and necrosis [12]. To gain a better understanding of this phenomenon and to identify key molecular effector molecules involved before, during and after the onset of ischemia, several animal models have been developed to mimic as closely as possible the alterations found during liver ischemia [13]. Among these experimental models, the left portal vein ligation (LPVL) model has been shown to accurately reproduce the phenotypical and functional alterations observed during liver ischemia [14, 15].

Anaerobic glycolysis or the *Pasteur effect* is an important cellular protective process that has been described both *in vivo* and *in vitro* [16]. This process leads to increased anaerobic glycolysis that promotes cell survival by maintaining ATP production and preventing membrane permeability transition (MPT) despite reduced oxygen levels [17]. Treatment with amino acids (glycine or alanine) [18] or with fructose [19] during ischemic events has been shown to increase cell survival. However, these protective mechanisms mainly target glutathione and the glycolytic pathway, which is only the first step of the four major pathways leading to the production of ATP.

Mitochondrial dysfunction and ATP depletion have also been shown to play a pivotal role in ischemia-induced liver injury [20]. However, in a rat experimental model, a 50% drop in inspired O_2_ did not lead to a significant decrease in adenosine tri-phosphate (ATP) levels in the liver despite a significant decline in hepatic hemoglobin oxygen saturation [21]. This strongly suggests that while decreased oxygenation of liver cells occurs during ischemia, it is not necessarily associated with reduced metabolic activity. Therefore, it remains unclear how both glycolytic and mitochondrial activities are affected during liver ischemia.

The aim of this study was to investigate whether the protection of hepatocytes observed early during the ischemic insult could originate from metabolic reprogramming and to discriminate the contribution of the glycolytic and mitochondrial pathways in maintaining metabolic homeostasis. Using high-performance liquid chromatography (HPLC) and high-resolution nuclear magnetic resonance (NMR) spectroscopy, we thoroughly characterized the energy metabolism of liver cells using the LPVL experimental model of ischemia [15]. Assessment of the liver bioenergetic status revealed a preserved ATP/ADP ratio and energy charge level, but also increased amounts of unlabeled succinate and glutamate, and greater enrichments in ^13^C-alanine and ^13^C-lactate. Therefore, these results strongly suggest that mitochondrial metabolism can synergistically couple with the *Pasteur Effect* during early ischemic events to prevent or delay hepatocyte injury.

## Materials and methods

### Materials

Deuterium oxide (D_2_0) and [U-^13^C]glucose were purchased from Cambridge Isotopes (Andover, MA, USA). Methanol and polyethylene tubing (PE-50) were obtained from Fisher Scientific (Ottawa, ON, Canada). 4–0 Suture silk was bought from Ethicon Inc. (NJ, USA). 3M Vetbond tissue adhesive was obtained from CDMV Inc. (Saint-Hyacinthe, QC, Canada). Unless stated otherwise, products and chemicals were purchased from Sigma-Aldrich (St-Louis, MO, USA).

### Animals

Sprague-Dawley male rats (4–8 weeks old) were purchased from Charles-River (Saint-Constant, QC, Canada) and were fed *ad libidum* with normal chow in vented cages. All procedures were performed in accordance with Canadian Council on Animal Care and approved by the *Comité institutionnel de protection animale du CHUM*.

### Left portal vein ligation (LPVL)

To minimize the effects of metabolite dilution by unlabeled glucose from glycogen, the animals were fasted overnight before surgery. Male Sprague-Dawley rats were anesthetized and placed on a temperature-controlled heating pad throughout surgery. Briefly, a laparatomy was performed, and the stomach and intestines were gently moved dorsally away from the liver. The portal vein was separated from the hepatic artery and bile duct before ligation with 4.0 braided silk between the junction leading to the left and median lobes and the portal branch supplying the right lobes of the liver [14, 15]. The surgical site was closed and the rat allowed to recover. 45 min prior to the end of the LPVL experiment, the animal was placed under isoflurane anesthesia, and PE-50 cannulae were introduced into the right jugular vein and left carotid artery. [U-^13^C] glucose [111 mg/mL (0.12 μg/min/g rat)] was infused into the right jugular vein for 45 min prior to the end of an LPVL period. The concentration and rate of infusion was chosen based on preliminary experiments to allow for steady-state conditions while minimizing hyperglycemia. At the chosen time, the liver was rapidly excised, weighed, the left and median lobes separated; then several slices were fixed for staining and the remaining liver was flash frozen in liquid nitrogen. Blood was obtained at sacrifice in order to collect serum. Sham surgeries were performed through a laparotomy with surgical manipulations required to isolate the left branch of the portal vein without placement of the ligature. Cannulations and [U-^13^C]glucose infusions were undertaken as in LPVL animals.

### Measurement of oxygen tension and blood flow in the liver

Tissue pO_2_ and relative local blood flow in the left liver lobes were measured with a combined oxygen-sensitive ruthenium red microelectrode and laser Doppler flow probe (Oxylite 2000, Oxford Optronix, Oxford, United Kingdom). The rats were anesthetized with isoflurane, intubated, and ventilation was adjusted in a pressure-controlled ventilator to achieve normocapnia and normoxia as quantified by blood gas analysis (ALB 500 and OSM 3 Radiometer, London Scientific, London, ON, Canada). The tail artery was cannulated (Angiocath 24 G, BD Medical, Oakville, ON, Canada) to continuously monitor blood pressure with a pressure transducer (Utah Medical Products, Midvale, UT, USA). A laparatomy was performed, and a loose ligature was placed around the left portal vein. Small plastic loops (to limit movement of the liver during measurement periods) were glued to the left lateral lobe, approximately 1 cm from the hilum with 3M Vetbond veterinary adhesive. Oxygen and flow probes were inserted approximately 1.5 mm below the capsule and adjusted with a stereotaxic apparatus so that stable tissue oxygen tension of 18–30 mmHg was achieved for at least 20 min prior to ligation. Tissue oxygen tension and relative blood flow were monitored continuously before and for 3 h after ligation. The invasive nature of this monitoring did not allow us to perform accurate and reliable measurements beyond 3 h after ligation [22]. At the end of the experiment, correct placement of the probes was confirmed by epinephrine administration (which caused a rapid increase in both pO_2_ and blood flow) and by visual inspection.

### Caspase 3 activity

As described previously [23], sections of frozen tissue were lysed in ice-cold caspase lysis buffer (10mmol/L HEPES [pH 7.4], 5mmol/L MgCl2, 42mmol/L KCl, 0.1mmol/L EDTA, 0.1% 3-[(3-cholamidopropyl) dimethylammonio]-2-hydroxy-1-propanesulonate (Calbiochem, San Diego, CA, USA), 0.1% Triton X-100, 1mmol/L dithiothreitol (DTT), 1mmol/L phenylmethyl-sulfonyl fluoride, 10μg/mL leupeptin, 10μg/mL aprotinin, 10μg/mL soybean trypsin inhibitor, and 100μmol/L benzamidine). The lysates were then centrifuged for 10 min at 14000g. Protein content was evaluated by the Bradford method and a fluorimetric Ac-DEVD-AMC cleavage assay was performed. The reaction contained 40μL of liver lysate (100μg of protein) and 50μL of reaction buffer (2×; 100mmol/L HEPES [pH 7.2], 200mmol/L NaCl, 2mmol/L EDTA, 20% sucrose, 0.2% 3-[(3-cholamidopropyl) dimethylammonio]-2-hydroxy-1-propanesulonate, 20mmol/L DTT). Plates were warmed to 37°C in a pre-heated Synergy HT spectrofluorometer platereader (Biotek, Winooski, VT, USA) and 10μL of ac-DEVD-AMC [100μmol/L] caspase 3 substrate was added to the reaction. Cleavage activity of caspase 3 was evaluated using 380nm/440nm excitation and emission wavelengths respectively (Synergy HT spectrofluorometer platereader, Biotek, Winooski, VT, USA). Maximal cleavage rate (Vmax/s) was calculated and caspase 3 activity was evaluated using calibration curves linking Vmax/s to units of activated recombinant caspase 3.

### ALT measurement

Quantitative determination of ALT levels was performed on serum samples using a multiparameter automatic biochemical analyser (Synchron LX System, Beckman Coulter, Mississauga, ON, Canada) by the Biochemistry Department of CHUM.

### Histological analysis

#### Hematoxylin-phloxine-saffron staining (HPS)

Formalin-fixed liver samples obtained at the time of sacrifice were set in paraffin blocks, sliced (4 μm sections) and stained with hematoxylin-phloxine-saffron by the Pathology Department of CHUM. Microphotographs were taken with a Carl-Zeiss Axioplan 2 microscope (Göttingen, Germany) at 20-40x magnifications using the Northern Eclipse 6.0 software (Empix Imaging, Mississauga, ON, Canada).

#### Hypoxia-inducible factor 1α staining (HIF-1α)

Slides were prepared from paraffin-embedded tissue from the left lateral lobe of the liver. Following standard deparaffinization procedures, the slides were subjected to heated antigen retrieval buffer containing citrate (10 mM) and Tween 20 (0.05%) at pH 6.0. Slides were then incubated with iso-osmotic TRIS-HCl (50 mM), NaCl (50 mM) buffer containing 0.05% Tween 20 for 10 min. Immunohistochemistry was performed using the EnVision+ Dual Link System-HRP (DAB+) immunohistochemistry kit (Dakocytomation, Mississauga, ON, Canada). Mouse anti-HIF-1α antibody (Calbiochem Inc, San Diego, CA, USA) was used at a dilution of 1:100. Microphotographs were taken with a Carl-Zeiss Axioplan 2 microscope (Göttingen, Germany) at 10x magnification using the Northern Eclipse 6.0 software (Empix Imaging, Mississauga, ON, Canada).

### Nuclear magnetic resonance (NMR) sample preparation and analysis

NMR sample preparation and analysis were performed as previously reported [24]. Briefly, frozen liver samples were grounded into a fine powder over liquid nitrogen. The powder was quickly poured into a pre-cooled (on ice) potter-elvehjem glass tube and 3 mL of ice-cold 5% perchloric acid (PCA) was added. The samples were homogenized on ice at 3000rpm with a motorized potter-elvehjem teflon homogenizer. The homogenized portion was decanted into a separate tube, and the residual powder was homogenized again in another 2 mL of 5% PCA. Both homogenates were combined and centrifuged at 4000g (4°C) for 20 min. The supernatant (containing water-soluble hydrophilic metabolites) was neutralized on ice to pH 7.0 with KOH. Precipitated potassium perchlorate was separated by centrifugation at 4000g for 20 min (4°C). The supernatant was placed in a round-bottomed glass flask and frozen in liquid nitrogen. This water-soluble liver extract was then lyophilized overnight. The lyophilisate was then re-dissolved in 600 μL D_2_0 and placed in a 5 mm glass NMR tube. The pH in each NMR sample was adjusted with NaOH until a stable value of 6.8 was reached prior to NMR analysis. NMR experiments were performed on a DRX 600 (600 MHz) Bruker spectrometer. ^1^H-NMR spectra were obtained with a 5 mm H,C,N inverse triple-resonance probe with a flip angle of 40°, a repetition time of 15 sec, a spectral width of 7,183 Hz and a data size of 16 K. Chemical shifts were referenced to lactate (1.33 ppm). ^13^C-NMR spectra were recorded with a C,H dual-probe, with a flip angle of 27°, a repetition time of 2.5 sec, composite pulse decoupling with WALTZ-16, and a spectral width of 47,619 Hz.

Unlabeled alanine, glucose, glutamate, lactate and succinate concentrations were analyzed from ^1^H-NMR spectra of PCA extracts using (trimethylsilyl) propionic-2,2,3,3d4-acid as external standard. ^13^C-labeled glucose, alanine and lactate were quantified from ^1^H-NMR spectra of liver extracts by integrating the satellite peaks caused by heteronuclear coupling between adjacent hydrogen and the glucose ^13^C-1, alanine ^13^C-3 and lactate ^13^C-3. ^13^C-fractional enrichment of these metabolites was calculated by dividing the satellite peak integral with the integral for protons adjacent to ^12^C.

^13^C-labeled succinate and glutamate were analyzed from the fractional enrichments and concentrations of lactate or alanine (as internal standards) were analyzed from ^1^H-NMR spectra. Percentage of ^13^C-enrichments of other metabolites was quantified from signal intensities in ^13^C-NMR spectra after correcting for relative nuclear overhauser enhancement, saturation effects and natural abundance of ^13^C.

### HPLC analysis

Hepatic ATP, ADP, AMP and Glutamine levels were measured from the left and median ischemic liver lobes. Liver samples weighing approximately 100 mg were homogenized in 0.5 mL of ice cold, nitrogen-saturated acetonitrile CH_3_CN + 10 mM KH_2_PO_4_ (pH7.4) (75:25). The solution was centrifuged for 5 min at 4°C and 14000g. The supernatant was transferred into a separate tube and extracted with 0.25 mL of chloroform and centrifuged at 14000g for 10 min at 4°C. The aqueous phase was carefully separated from the hydrophobic phase and placed into a separate tube. For HPLC analysis, 50 μL of sample was applied to a Phenomenex reverse phase column (Gemini^™^ C18, 5 μm, 250x4.6 mm) maintained at 18°C and connected to an Agilent 1100 HPLC system with the proprietary inline degasser, quaternary pump, autosampler, column thermostat, and diode array detector. Elution was performed at a constant flow rate of 1.2 mL/min using a stepwise gradient from mobile A (10 mM KH_2_PO_4_, 10 mM tetrabutylammonium hydroxide (TBAH) and 0.125% methanol) to B (100 mM KH_2_PO_4_, 2.8 mM TBAH and 30% MeOH). Detection was carried out at 267 nm. Metabolite content is expressed in nanomole per gram of liver tissue and the energy charge was calculated using this formula: ([ATP] + ½[ADP]) / ([ATP] + [ADP] + [AMP]) [25].

### Statistical analysis

All data represent the values of at least three independent experiments. Data are expressed as means ± standard error (SEM) and were analyzed with GraphPad Prism7 software. Differences between groups were analyzed using the analysis of variance (ANOVA) test, student *t*-test and Tukey post-test for multiple comparisons. A *P* value below 0.05 was considered significant (* *= P*<0.05, ** *= P*<0.01, *** *= P*<0.001). All statistical tests were two-sided.

## Results

### Lack of hepatocyte cell death during the early phases of liver ischemia

**Fig 1:**
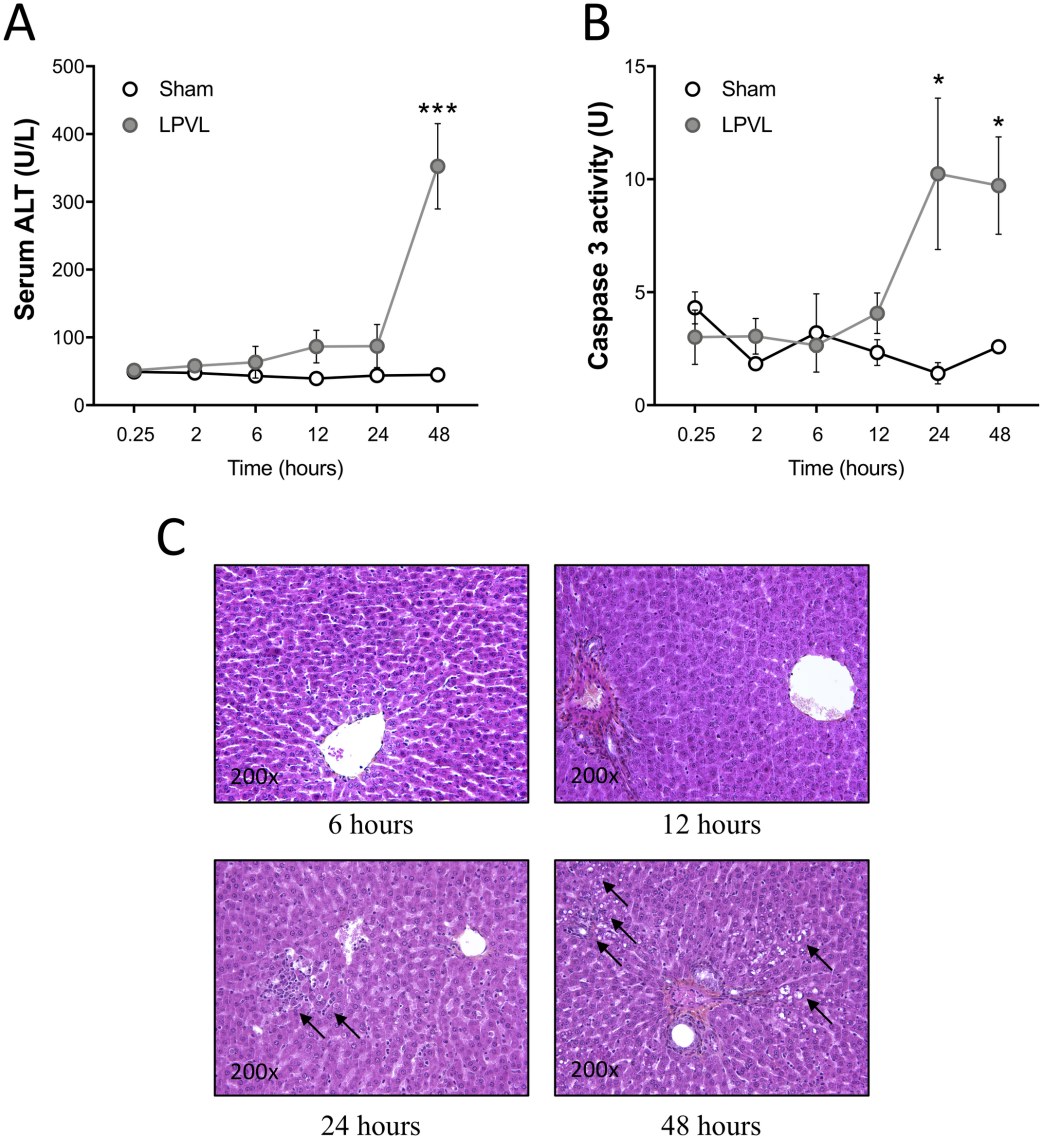
Delayed hepatocyte injury after induction of ischemia. (A) Evaluation of ALT serum levels and (B) quantification of caspase 3 activity in sham and LPVL-treated rats over a period of up to 48 h following induction of ischemia. (C) Representative microphotographs of HPS staining of the left liver lobe of LPVL-operated rats over a period ranging from 6 to 48 h following ischemia. Liver injury was assessed by the histological evaluation of necrosis. Values are ±SEM of 3–8 different animals. (*P<0.05, ***P<0.001).

Ischemia can lead to hepatocyte cell death but the sequence of events leading to it needs to be defined. To characterize this phenomenon, we first looked at ALT serum levels in both LPVL and sham-operated rats, for times ranging from 0.25 to 48 h after the onset of ischemia. While the control group maintained normal ALT levels irrespective of the time after surgery, LPVL animals showed increased levels of ALT only 48 h after LPVL (*P*<0.001) (Fig 1A). In line with these observations, the tissular activity of Caspase 3 in ischemic tissues was only found to be increased 24 and 48 h after LPVL (*P*<0.05) (Fig 1B). To monitor the ischemia induced by LPVL in our model, the total relative blood flow in the left liver lobes was measured and found to be decreased to approximately 50% (*P*<0.05) over a period of 90 min after surgical ligature (S1A Fig). Flow thereafter remained constant at 50% for the rest of the analysis. Oxygen tension in the left lobes was measured for 20 min prior to ligation and for the first 150 min post-ligation. LPVL caused a gradual decrease in tissue pO_2_ over a period of 180 min to approximately 40% of initial pO_2_ (from 19.5±8.72 mmHg to 8.03±4.64 mmHg) (S1B Fig). Liver sections from LPVL-treated animals showed apoptotic bodies and/or cell vesicles, hallmarks signs of cell death, only 24 and 48 h after LPVL while no histological evidence of liver injury could be observed 6 and 12 h after LPVL (Fig 1C). HPS staining of the right liver lobes (non-ligated) showed no sign of cell death during the 48-h period although it showed signs of vacuolization at 24 h, a characteristic of hepatocellular regeneration (S1C Fig). Therefore, hepatocyte injury is delayed after LPVL-induced ischemia.

### Glucose uptake levels under hypoxic condition

**Fig 2:**
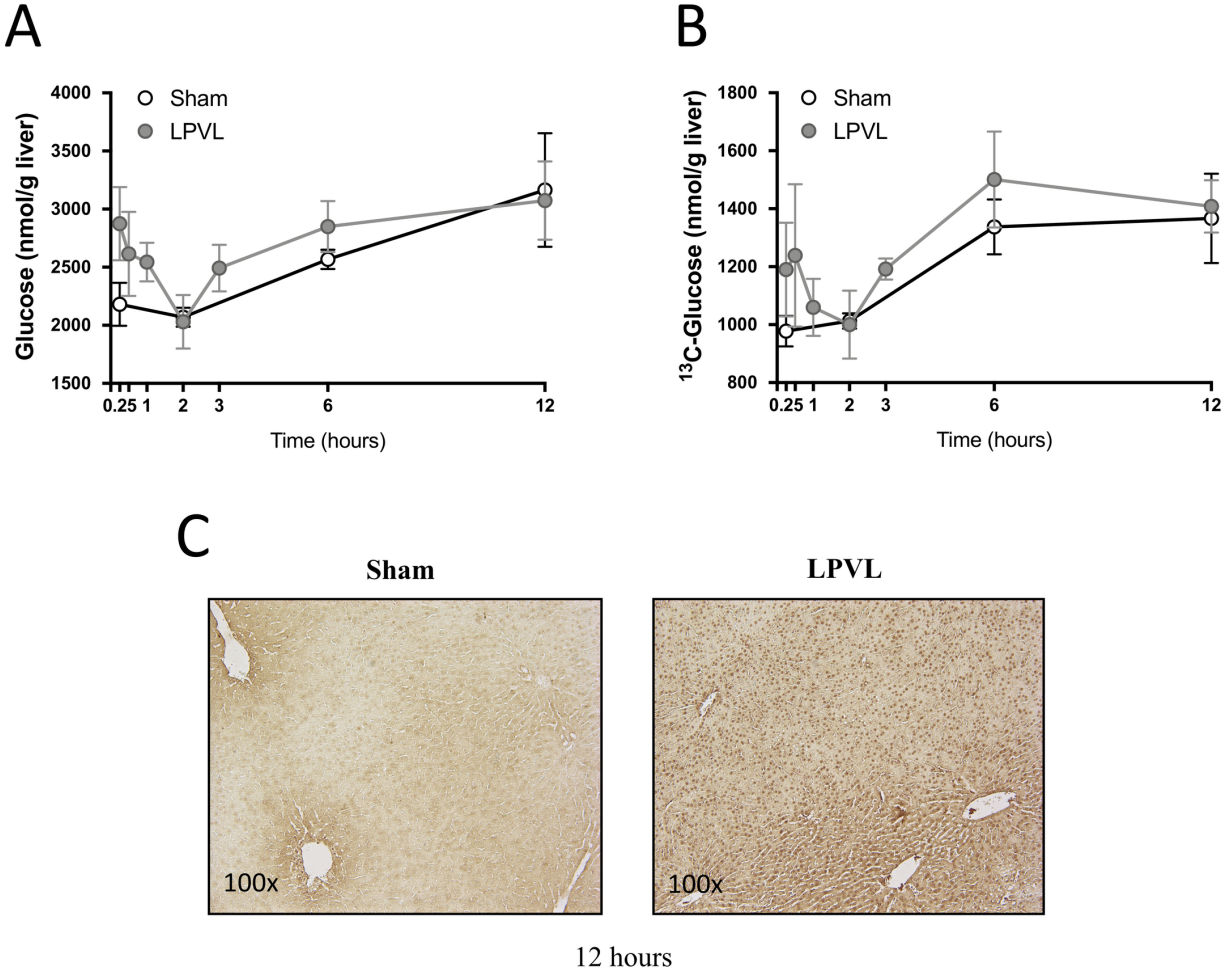
Time course of total and ^13^C glucose levels during hypoxia. (A) Total levels of hepatic glucose of sham-operated (filled white circles) and LPVL-treated (filled grey circles) rats. (B) Levels of ^13^C glucose in sham (filled white circles) versus LPVL-treated (filled grey circles) rats. [U^-13^C] glucose was infused for 45 min after the indicated times. (C) Representative microphotographs of Hypoxia inducible factor 1-α (HIF 1-α) staining of the left liver lobe of LPVL-treated animals 12h after ischemia. Slides were stained with Mayer’s Hematoxylin and mouse-anti-HIF-1α. Values are expressed as the mean ± SEM of 3–10 animals per group.

Hepatic glucose concentrations were measured 0.25 to 12 h after LPVL. LPVL animals showed similar hepatic glucose concentrations to sham-operated animals (3073±336 vs 3164±489 nmol/g liver, respectively, 12 h after surgery) (Fig 2A). Similarly, measurements of the uptake of infused ^13^C-glucose were strikingly similar between LPVL animals and sham-operated animals (1408±90 vs 1367±154 nmol/g liver, respectively, 12 h after surgery) (Fig 2B). In line with these observations, the enrichment of ^13^C-glucose in the liver of LPVL- and sham-operated rats were not significantly different up to 12 h after surgery. HIF-1α, a protein induced by tissue hypoxia, can upregulate genes involved in glucose metabolism [26, 27]. LPVL caused an increase in the expression of HIF-1α 12 h after surgery (Fig 2C). HIF-1α staining was strong and uniform throughout the liver lobule with a characteristic nuclear pattern in the ligated liver lobes in comparison to sham controls where immunostaining was mild, cytoplasmic and largely confined to the pericentral area (Fig 2C). Therefore, hepatocytes can maintain normal glucose uptake even under hypoxic condition and loss of portal flow.

### Impact of LPVL on glucose metabolism

#### Energy charge

**Fig 3:**
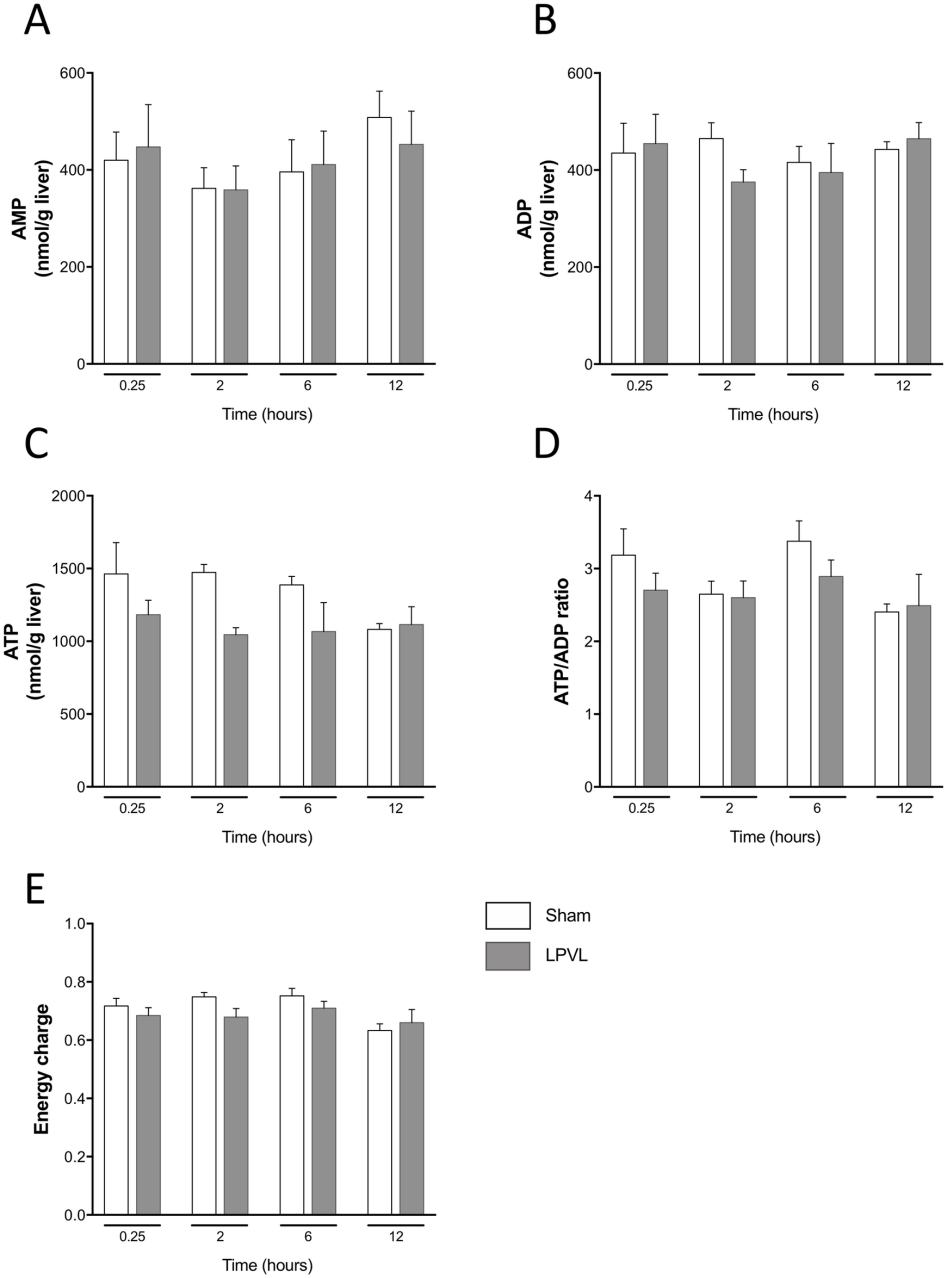
ATP/ADP ratio and energy charge levels in the left lateral lobe of sham and LPVL-treated rats. Hepatic (A) AMP, (B) ADP, (C) ATP, (D) ATP/ADP ratio and (E) energy charge of sham and LPVL-operated rats over a period ranging from 0.25 to 12 h following ischemia. Values are expressed as the mean ± SEM of 3–8 animals.

To assess if the ability of hepatocytes to maintain glucose uptake, despite ischemia, is also reflected in their capacity to produce/maintain energetic metabolites, we measured the tissue contents of AMP, ADP and ATP by HPLC and calculated the energy charge for each animal. Concentrations of AMP, ADP and ATP were not significantly different irrespective of the time after surgery (Fig 3A–3C). While the ATP/ADP ratio fluctuated slightly 2 and 6 h after LPVL, no significant difference was observed between control and LPVL-treated animals for every time point studied (Fig 3D). Moreover, no significant difference was observed over time in the calculated energy charge (([ATP] + ½[ADP]) / ([ATP] + [ADP] + [AMP])) within each group and between control and LPVL-treated animals (Fig 3E).

#### Mitochondrial metabolism

**Fig 4:**
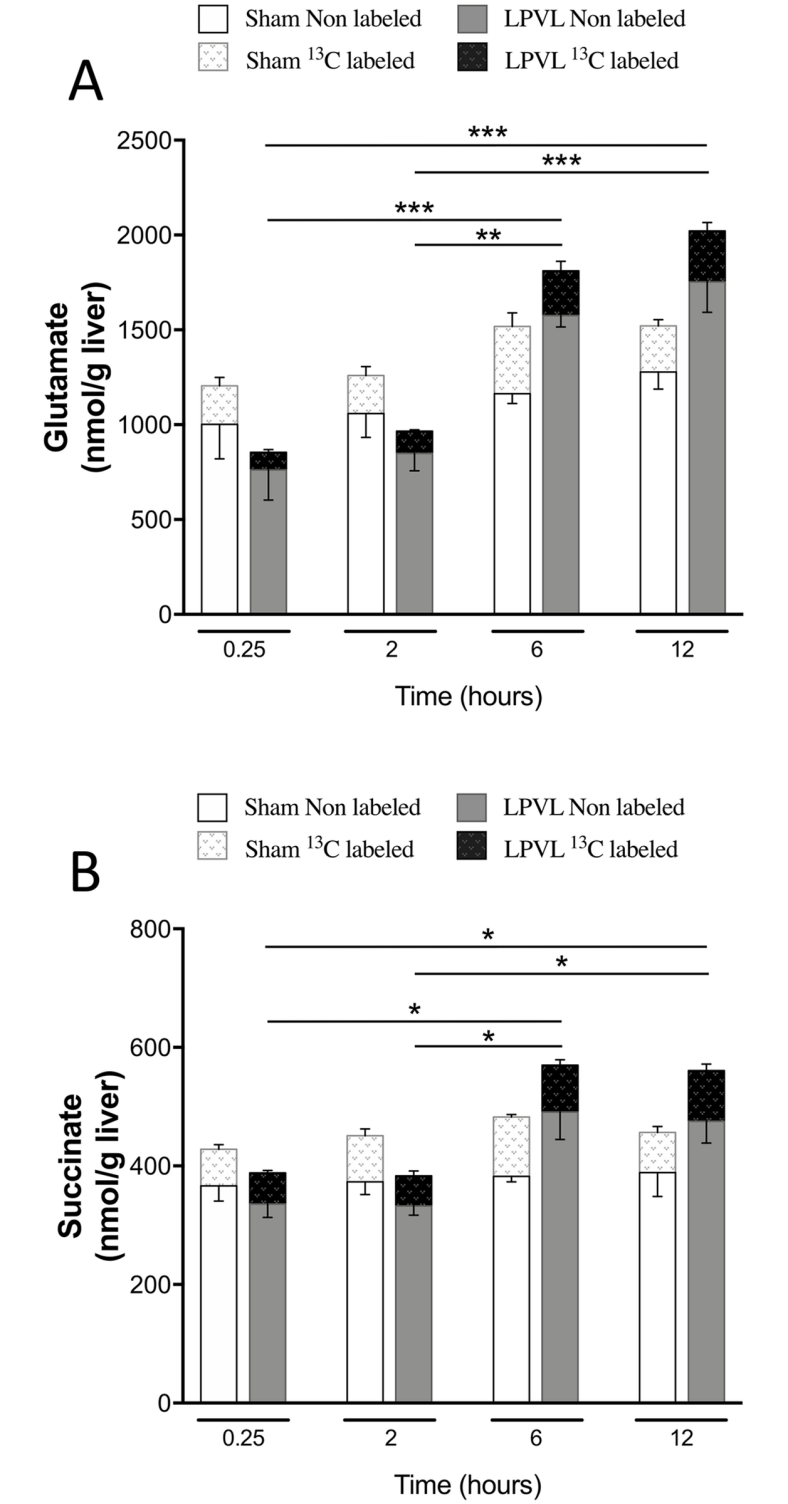
Time course of total and ^13^C-enriched hepatic glutamate and succinate in the ligated lobe following LPVL. Unlabeled levels of hepatic (A) glutamate and (B) succinate of sham-operated rats (open white squares) and LPVL-treated (filled grey squares) rats. ^13^C-enrichment of the metabolites: (A) glutamate and (B) succinate in sham (white patterned squares) and LPVL-treated (black patterned squares) rats. Values are expressed as the mean ± SEM of 3–6 animals per group. (*P<0.05, **P<0.01, ***P<0.001 when total glutamate and succinate levels were compared in (A-B)).

The preserved ATP/ADP ratio and energy charge level of ischemic hepatocytes could originate from enhanced mitochondrial metabolism, Krebs cycle activity and associated metabolite levels. While total glutamate level in LPVL-treated rats slightly increased 2 h after LPVL (966±95 vs 707±87 nmol/g liver, 2 h and 0.25 h after LPVL respectively), a more significant increase reaching approximately 60% was observed at later time-points (1810±103 and 2088±340 nmol/g liver respectively 6 and 12 h after LPVL) (*P*<0.01) (Fig 4A). Total succinate levels in the LPVL group increased by 32% 6 to 12 h after ligation (394±28, 376±15, 592±64 and 577±43 nmol/g liver respectively, 0.25, 2, 6 and 12 h after LPVL) (*P*<0.05) (Fig 4B). However, no increase in ^13^C-enrichment over time was found for both metabolites in the LPVL group (Fig 4A and 4B). Quantification of total glutamine content in the LPVL group also revealed increased levels 6 and 12 h after surgery (2475±304, 2499±136, 4165±483 and 4928±139 nmol/g liver respectively at 0.25, 2, 6 and 12 h) (*P*<0.05) (S2 Fig). No significant variation over time was observed in the sham control group in the concentrations of total glutamate, succinate and glutamine, but also in ^13^C-enrichments (Fig 4A and 4B and S2 Fig).

#### Glycolysis

**Fig 5:**
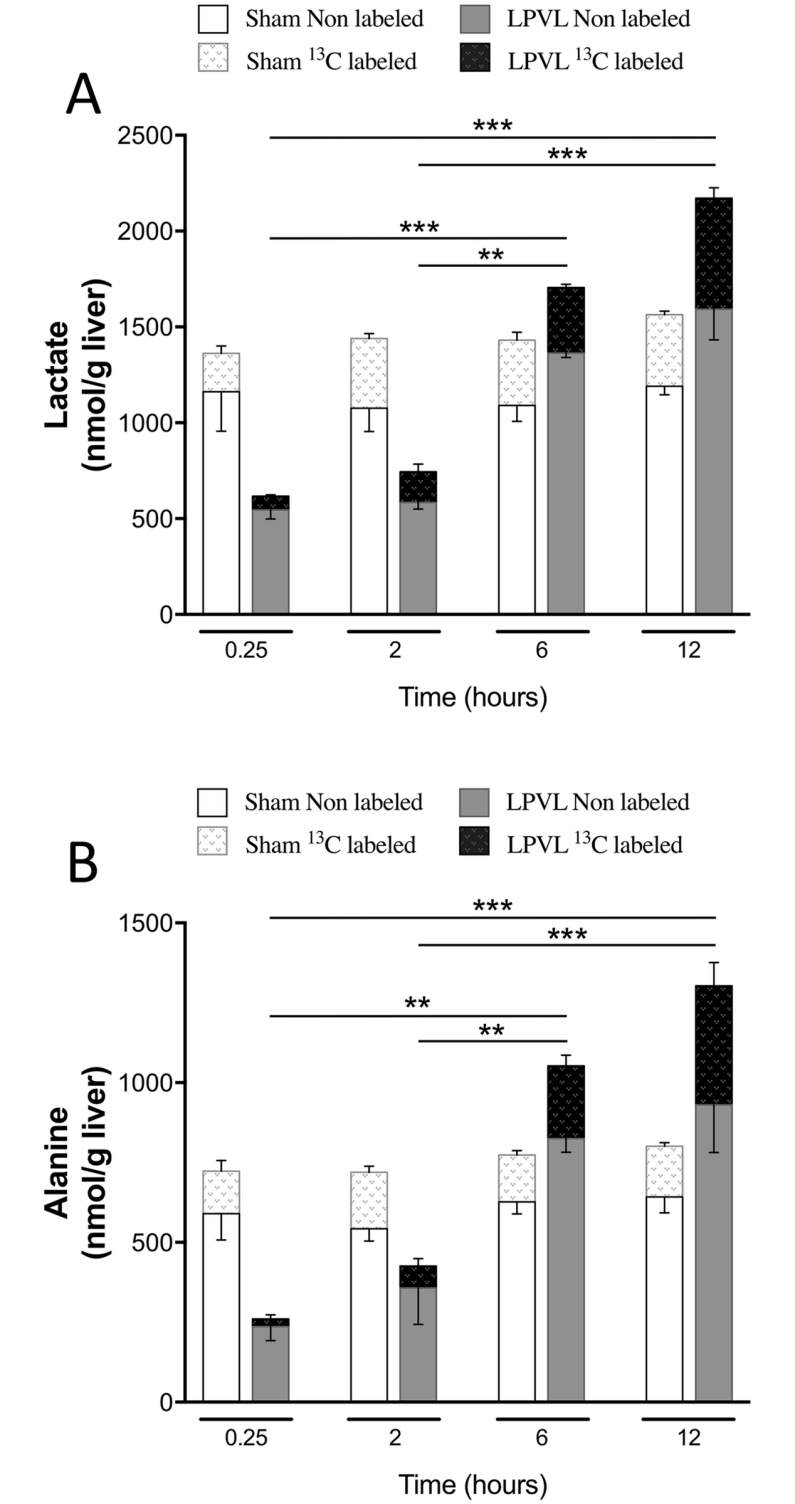
Time course of total and ^13^C-enriched hepatic lactate and alanine in the ligated lobe following LPVL. Unlabeled levels of hepatic (A) lactate and (B) alanine of sham-operated rats (open white squares) and LPVL-treated (filled grey squares) rats. ^13^C-enrichment of metabolites: lactate (A) and alanine (B) in sham (white patterned squares) and LPVL-treated (black patterned squares) rats. Values are expressed as the mean ± SEM of 3–5 animals per group. (**P<0.01, ***P<0.001 when total lactate and alanine levels were compared in (A-B)).

Since change in glycolysis and the synthesis of lactate and/or alanine could occur after LPVL, total and ^13^C-labeled lactate and alanine concentrations were measured. Total lactate level during ischemia increased slightly 2 h after LPVL (744±67 vs 616±54 nmol/g liver) (Fig 5A). However, total lactate level increased by 64% at later time points (1705±32 and 2283±214 nmol/g liver respectively 6 and 12 h after LPVL) (*P*<0.01) (Fig 5A). Similarly, the enrichment in ^13^C-labeled lactate, which was initially lower in the LPVL group, gradually increased over time by 45% (from 11±0.2% to 20±0.6% and 25±0.9% respectively 0.25, 6 and 12 h) (*P*<0.01) (Fig 5A). Total alanine content was slightly increased 2 h after LPVL (376±70 vs 253±52 nmol/g liver) while a significant 78% increase was observed beginning 6 h after LPVL (1174±32 and 1345±250 nmol/g liver respectively 6 and 12 h after LPVL) (*P*<0.01) (Fig 5B). ^13^C-labeled alanine enrichment was also initially lower in ischemic animals but gradually recovered and increased by 65% over time (from 6.7±3.5% to 19±1.7% and 23±4.4% respectively 0.25, 6 and 12 h) (*P*<0.05) (Fig 5B). Sham-operated animals maintained stable values for both metabolite concentrations and ^13^C-enrichments over time (Fig 5A and 5B).

## Discussion

The liver is central to glucose metabolism, producing substrates involved in several metabolic pathways [28, 29]. When there is a significant decrease in the blood flow, the liver is exposed to the risk of being deprived of sufficient substrates for energy generation [30]. The accumulation of toxic metabolites in the liver, such as lactate, requires an excess consumption of ATP to restore metabolite homeostasis [31]. This metabolic feature has been observed in particular in the setting of organ transplantation [7]. Actually, one of the most important aspects of liver transplantation is the ability of the liver to generate energy-rich substrates after transplantation, and failure to do so can lead to graft nonfunction [32, 33]. Thus, understanding the metabolic impact of liver ischemia on hepatocytes remains of paramount importance.

Using the LPVL rat model, we found that there is a lag phase of 24 h after the onset of ischemia before ALT levels and Caspase 3 activity start to increase and before any evidence of cell death can be observed. Thus, there is a “hepatoprotective” early phase that precedes liver injury where hepatocytes can survive despite the hypoxia secondary to the loss of portal flow. This delay could stem from an adaptation to these adverse conditions. Interestingly, there is an initial and transient elevation in both total and ^13^C-labeled hepatic glucose levels in LPVL animals: however, no significant difference is observed over time compared to controls. This initial increased uptake could have come from the hepatic arterial buffer response [34]. It is known that the cessation of portal flow is accompanied by a partially compensatory increase in arterial blood flow known as the hepatic arterial buffer response [34]. However, the increase in glucose was only transient in LPVL animals and rapidly returned to normal values 2 h after the onset of ischemia. Thus, it cannot explain the lack of injury observed during 24 h. Therefore, the ability of these hepatocytes to maintain glucose uptake and viability under hypoxic conditions requires metabolic reprogramming by these cells to maintain their functional integrity.

To study this metabolic reprogramming, we assessed hepatic energetic metabolites and observed that neither the ATP/ADP ratio nor the energy charge was modified in both groups for 12 h after surgery. Based on the fact that there is decreased portal flow and oxygenation during LPVL-induced ischemia, the preserved liver bioenergetic production and glucose uptake suggest an increased ability of these cells to metabolize glucose to compensate for the ischemia. Indeed, at 24 h, the ischemic liver shows significantly lower energy charge levels which coincide with elevation in ALT (data not shown); the stability of liver bioenergetic production found during the first 12 h following LPVL seems to be closely associated with the functional integrity and viability of hepatocytes. The importance of maintaining tissue energy levels has also been described in the myocardium where decreased ATP levels have been associated with the expansion of myocardial tissue necrosis during acute myocardial ischemia [35]. In addition, improving intrahepatic ATP levels in livers from old animals by administration of glucose has been shown to protect the liver against ischemic injury and restore the protective effects of ischemic preconditioning [36].

Mitochondrial metabolism and more particularly the Krebs cycle could be involved in the maintenance of this favorable metabolic profile. Interestingly, while total glutamate and succinate contents significantly increased over time in LPVL animals, no change was observed in the ^13^C-enrichments of glutamate and succinate. Since glucose metabolism occurs separately from this pathway, ^13^C from glucose cannot be incorporated into succinate and glutamate: this could explain the levels of ^13^C-glutamate and ^13^C-succinate we observed. However, this would suggest that 1) mitochondrial anaerobic metabolism is active in ischemic conditions and 2) there is an exogenous supply of Krebs cycle intermediates (increased total glutamate and succinate concentrations). This requires a gradual increase in one metabolite upstream of the glutamate pathway that is not part of the Krebs cycle. One of these upstream metabolites could be glutamine, the levels of which are higher 12 h after ligation compared to early time points. Under ischemia, mitochondrial glucose metabolism is reduced and an exogenous supply of glutamine could fuel the Krebs cycle and contribute to the glutamate and succinate pools of hepatocytes to preserve liver bioenergetics. In cancer, glutamine oxidation has been shown to maintain mitochondrial activity when glycolytic pyruvate transport was impaired [37]. Indeed, the absence of pyruvate import redirects glutamine metabolism to allow Krebs cycle activity to drive ATP production via oxidative phosphorylation and maintain cell survival [37].

These observations therefore raise the possibility of activating mitochondrial metabolism during ischemic injury. In ischemic hearts, a number of mitochondrial alterations have been described as a consequence of either ischemia or post-ischemic reperfusion such as reduced contractile activity and decreased membrane potential [38]. Nevertheless, NMR studies have also showed that oxidative phosphorylation might still be active in isolated ischemic hearts [39–41]. In line with these findings, isolated rat kidneys perfused under hypoxic conditions maintained ATP levels required to maintain renal function by the production of GTP and subsequent transphosphorylation of ADP into ATP [42]. In our study, it is important to note that LPVL induces hypoxic rather than anoxic conditions: this means that reduced but functional mitochondrial oxidative phosphorylation activity is very plausible. This could allow the generation of ATP molecules and contribute to the maintenance of liver bioenergetics.

The absence of change in the levels of glucose uptake (total and ^13^C-labeled) and energy charge could also stem from enhanced glycolytic activity. Consistent with the reduced oxygen availability and the well-described *Pasteur Effect*, total lactate and alanine contents gradually increased over time in our model. Similarly, ^13^C-labeled lactate and alanine levels also increased over time up to a maximum 12 h after LPVL. Therefore, as previously reported, glycolysis can compensate a decrease in mitochondrial potential by a rapid increase of its own activity to generate ATP, thus explaining the substantial glucose consumption observed by the liver of ischemic animals [43–45].

While anaerobic glycolysis has often been perceived as a metabolic escape pathway allowing cells to efficiently adapt to the lack of oxygen induced by ischemia, emerging evidences point towards a detrimental effects of its over activation [46]. Indeed, lactate accumulation leads to cellular acidification and also reduced active Ca^2+^ efflux [46]. Consequently, the re-uptake of calcium by the endoplasmic reticulum is reduced leading to an intracellular calcium overload. All these changes are accompanied by the opening of the mitochondrial permeability transition pores, a key factor in the activation of apoptosis [46]. Moreover, in the heart, these cellular modifications are accompanied by the activation of intracellular proteases which damage myofibrils and results in contracture band necrosis [47]. In liver diseases, including fulminant hepatic failure, lactic acidosis is associated with a poor prognosis [48, 49]. All these observations highlight the harmful effects of prolonged anaerobic glycolysis during ischemia, but also the possibility of increasing mitochondrial activity by the addition of Krebs cycle related metabolites. The addition of Krebs cycle intermediate could: 1) help maintain ATP levels since residual respiratory activity can occur under hypoxic conditions, and 2) counteract the anaerobic glycolytic flux by decreasing its activity and increasing ATP yield.

Therefore, hepatocytes could resist the harmful effect of ischemia for the first 12 h by modifying their metabolic activity through an increase in both the Krebs cycle and glycolytic metabolic activities. Unfortunately, these processes can only alleviate the negative impacts of hypoxia for a limited period of time: if normoxia is not reestablished, the irreversible impact of cell injury and subsequent cell death will occur, such as is observed after 12 h in our model. Our observations suggest that novel or improved metabolic strategies could be developed to prevent or minimize ischemic liver damage.

## Supporting information

S1 Fig LPVL experimental model of ischemia. (A) Blood flow was recorded in arbitrary blood perfusion units (BPU). (B) Hepatic tissue oxygen tension (shown as mmHg). (C) Representative microphotographs of HPS staining of the right liver lobe of LPVL-treated animals over a period ranging from 6 to 48 h following ischemia. Values are expressed as the mean ± SEM of 3–10 different animals. (*P<0.05).
(TIF)

S2 Fig Glutamine content profile in the liver. Hepatic content of glutamine evaluated over a period ranging from 0.25 to 12 h after ischemia. Values are expressed as the mean ± SEM of 3–6 animals. (*P<0.05, ***P<0.001).
(TIF)
