# Supplementary figures and images for: Upregulation of Krebs cycle and anaerobic glycolysis activity early after onset of liver ischemia
Source: PLoS One. 2018 Jun 14;13(6):e0199177. doi: 10.1371/journal.pone.0199177 (PMC6002017; doi:10.1371/journal.pone.0199177)

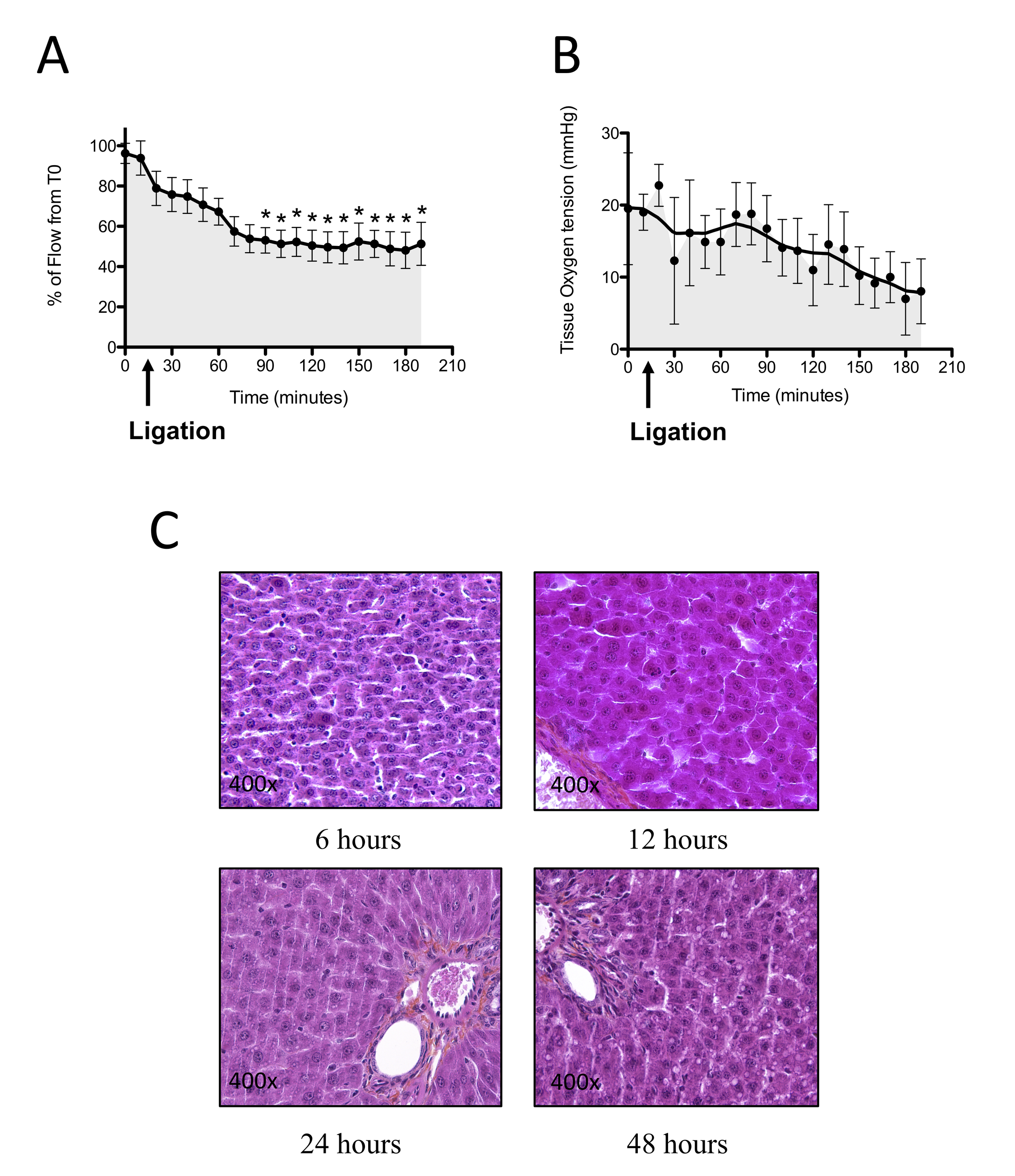

Supplement: S1 Fig — (A) Blood flow was recorded in arbitrary blood perfusion units (BPU). (B) Hepatic tissue oxygen tension (shown as mmHg). (C) Representative microphotographs of HPS staining of the right liver lobe of LPVL-treated animals over a period ranging from 6 to 48 h following ischemia. Values are expressed as the mean ± SEM of 3–10 different animals. (*P<0.05). (TIF) [file pone.0199177.s001.tif]

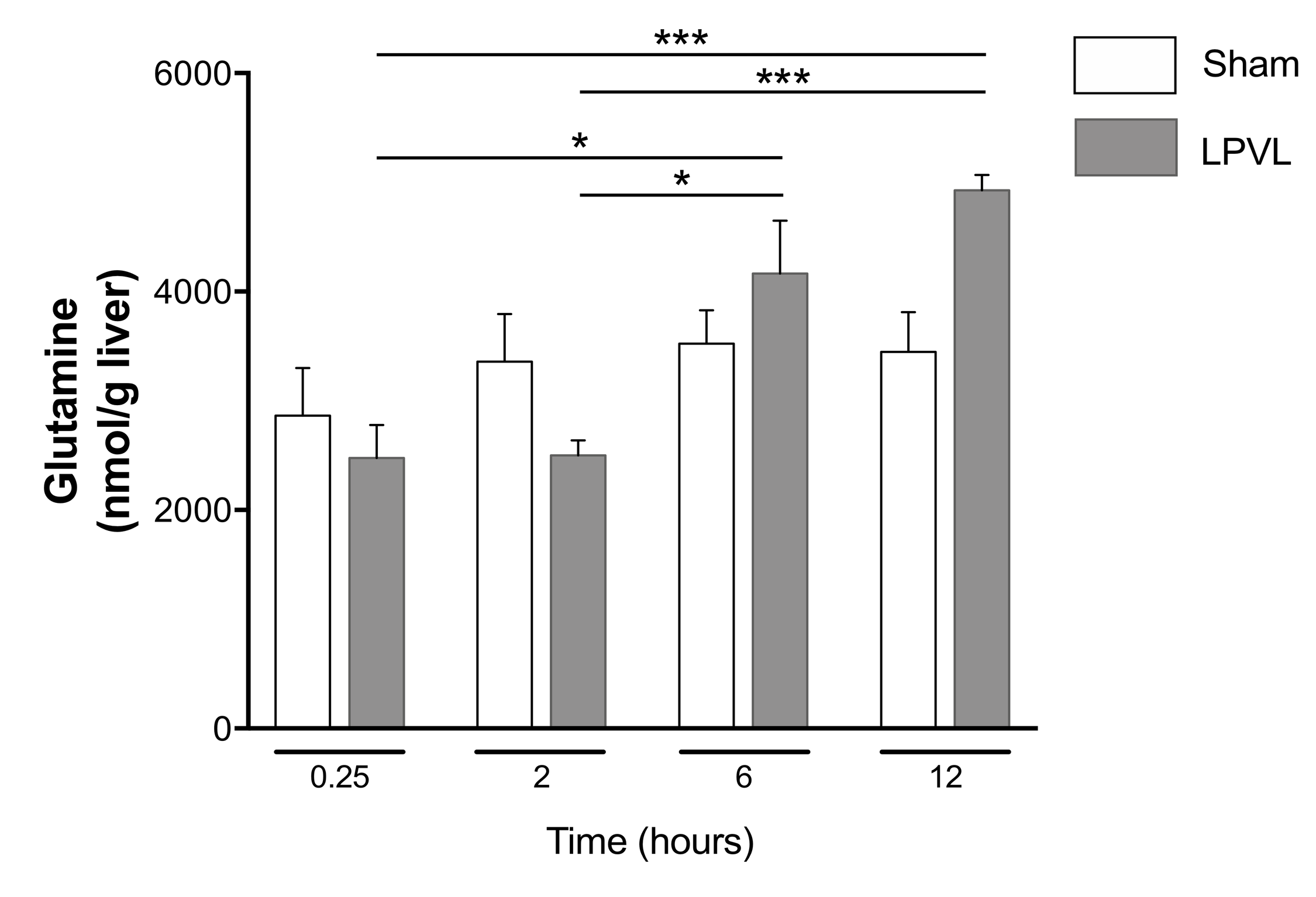

Supplement: S2 Fig — Hepatic content of glutamine evaluated over a period ranging from 0.25 to 12 h after ischemia. Values are expressed as the mean ± SEM of 3–6 animals. (*P<0.05, ***P<0.001). (TIF) [file pone.0199177.s002.tif]
